# Lower Extremity Arteriovenous Fistula Mimicking Deep Venous Thrombosis: A Case Report

**DOI:** 10.7759/cureus.20690

**Published:** 2021-12-25

**Authors:** Abdullah Alwohaibi, Nashwan Alattab, Mohammed AlSheef

**Affiliations:** 1 Internal Medicine, King Fahad Medical City, Riyadh, SAU; 2 Vascular Surgery, King Fahad Medical City, Riyadh, SAU; 3 Medicine, King Fahad Medical City, Riyadh, SAU

**Keywords:** unilateral leg swelling, interventional radiology guided embolization, lower extremity dvt, arteriovenous fistula, deep venous thrombosis (dvt)

## Abstract

Deep venous thrombosis (DVT) is the most common cause of unilateral lower limb swelling. Common differential diagnosis includes superficial thrombophlebitis and ruptured Baker's cyst. DVT is one of the most common complications diagnosed following lower extremity orthopedic surgery. However, many less frequent causes are often easily overlooked. Here we present a case of a 65-year-old man with a previous hip replacement who developed left-sided progressive leg swelling for years, which was managed initially with anticoagulation for provoked DVT and with compression stockings for post-thrombotic syndrome with no improvement. There was arterialization and spectral broadening of the venous waveform in the Doppler study. Computed tomography angiogram of the lower limbs showed evidence of arteriovenous fistula (AVF) with opacification of the deep left leg veins. Angioplasty and embolization of the fistula resulted in the resolution of leg swelling. We also discussed similar cases found in the literature. AVF needs to be considered in patients presenting with unilateral leg swelling following lower extremity orthopedic surgery.

## Introduction

Lower extremity deep venous thrombosis (DVT) is one of the most common complications diagnosed following lower extremity orthopedic surgery [1]. Venous thromboembolism occurs in one out of 40 patients undergoing internal fixation after hip fracture [2]. Rarely, arteriovenous fistula (AVF) can complicate orthopedic surgical procedures, causing symptoms similar to DVT [3]. We report a case of AVF masquerading as DVT.

## Case presentation

**Figure 1:**
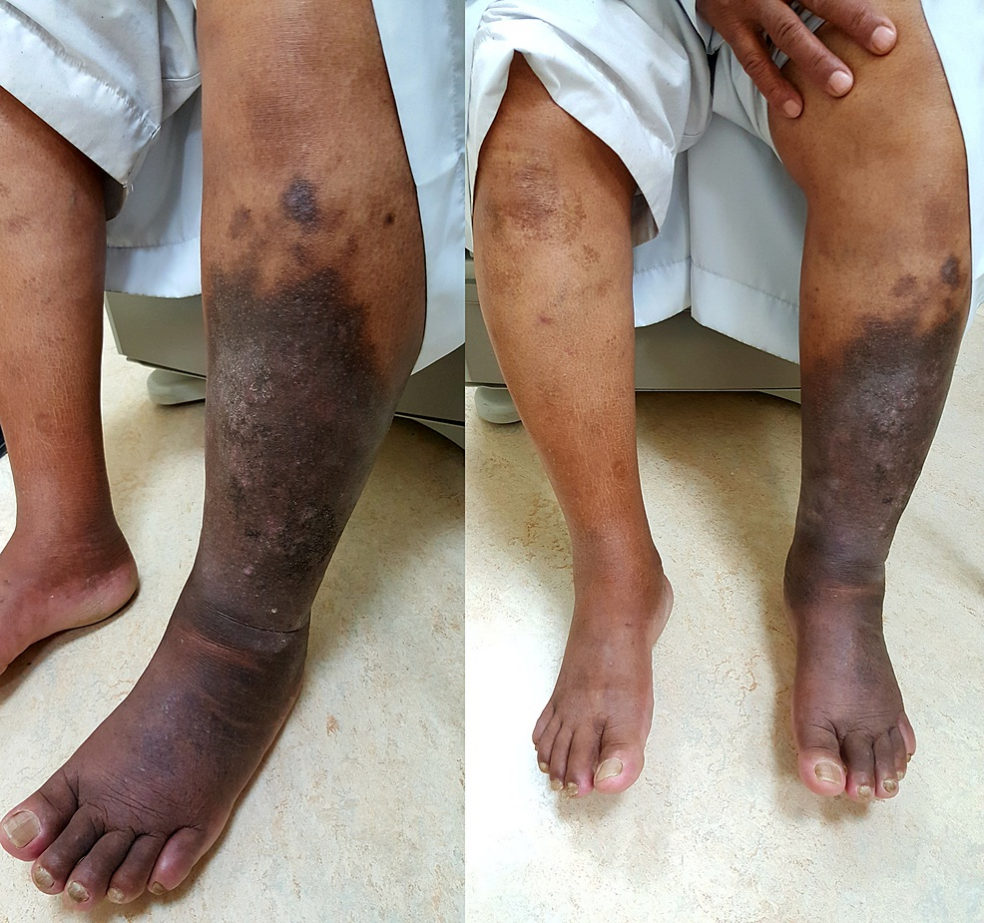
The appearance of the lower limbs on initial presentation showing left leg swelling, marked pigmentation, and lipodermatosclerosis

**Figure 2:**
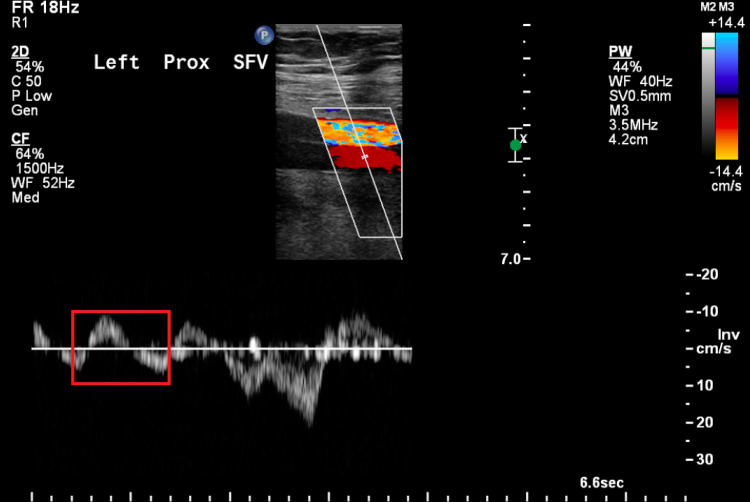
Left proximal superficial femoral vein Doppler showing arterialization of the waveform. Normally, lower extremity vein Doppler shows phasic non-pulsatile waveform. Red rectangle indicates biphasic pulsatile waveform, i.e., arterialization of the waveform.

**Figure 3:**
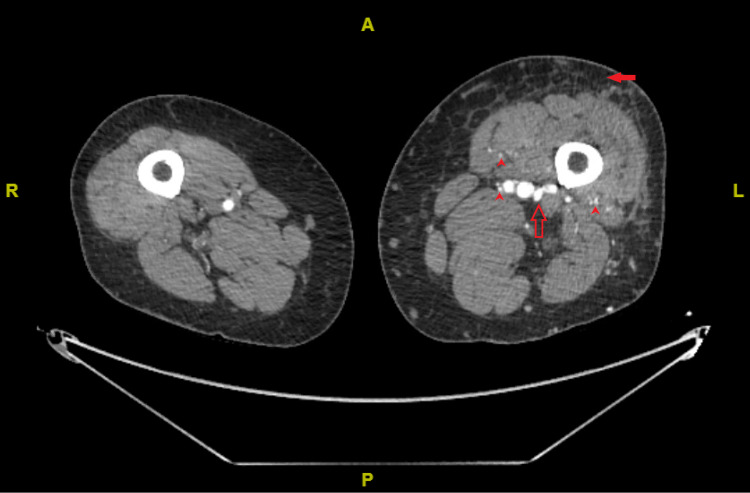
Computed tomography angiogram of the lower limbs showing leg asymmetry with opacification of the femoral veins in the arterial phase with substantial subcutaneous edema and formation of collaterals. Red-filled arrow indicates subcutaneous edema, red empty arrow indicates opacification of the femoral veins, and red arrowheads indicate opacification of venous collaterals.

**Figure 4:**
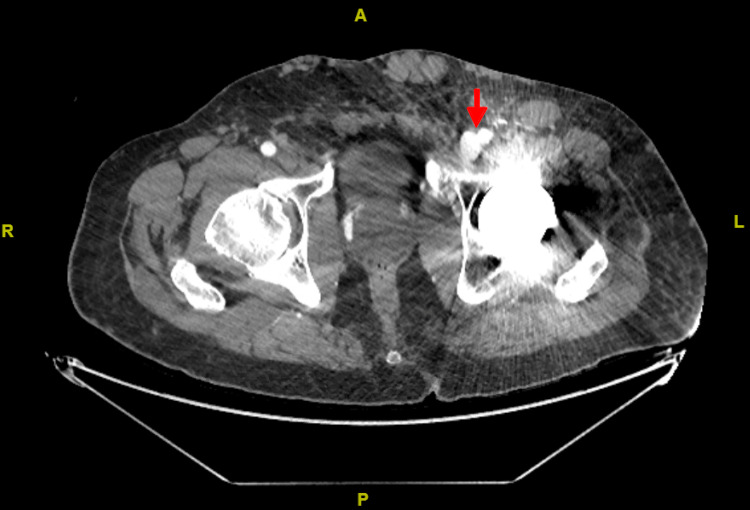
Computed tomography angiogram of the lower limbs showing opacification of the left common femoral vein at the level of the femoral head. Red arrow indicates opacified left common femoral vein at the level of the femoral head.

**Figure 5:**
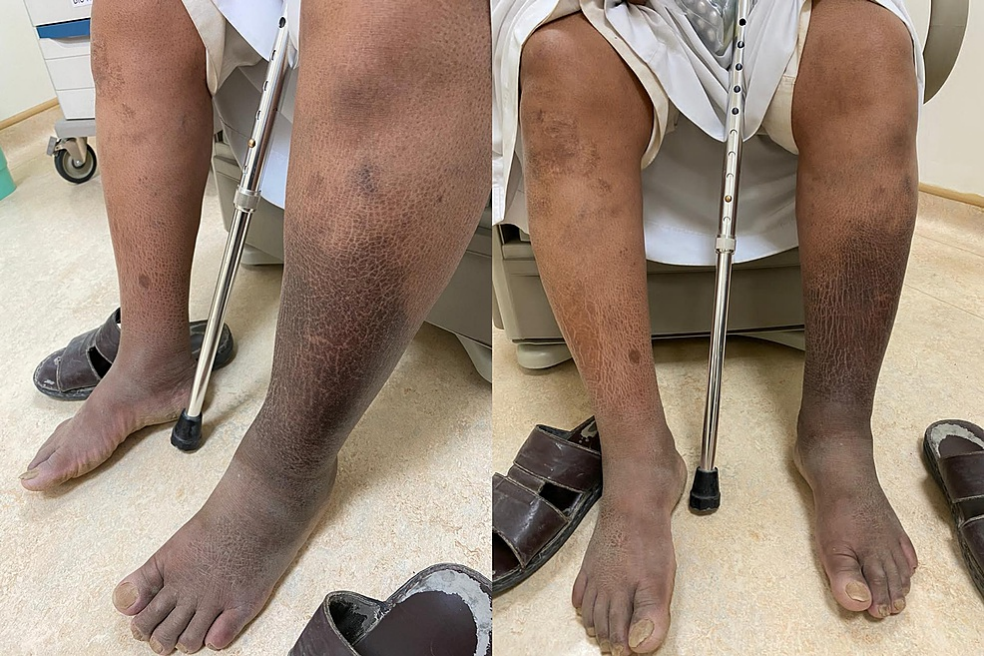
The appearance of the left leg following successful embolization and coiling of the arteriovenous fistula. It shows significant improvement in the swelling.

A 65-year-old man with well-controlled type 2 diabetes mellitus and hypertension was referred to our clinic for worsening left leg swelling for one year. He underwent left hip replacement for hip fracture three years before his presentation. He was diagnosed with left leg DVT and treated with oral anticoagulants for three months following his surgery. There was no clear evidence of DVT at that time on review. On examination, his left leg was grossly larger than its counterpart. There was diffuse pitting edema, pigmentation, and lipodermatosclerosis. There were no ulcerations, and pulses were present (Figure 1). He did not have signs of heart failure. We investigated with ultrasonography of the affected limb, and it showed patent and compressible deep veins with an arterialized waveform on Doppler (Figure 2); common femoral vein Doppler showed spectral broadening, indicating turbulent flow. Lymphoscintigraphy revealed no lymphatic obstruction. Computed tomography angiogram of the lower limbs revealed opacification of the left leg venous system in the arterial phase and abnormal vascularity with collaterals (Figures 3, 4). The patient was started on daily compression stockings during work-up with no benefit. The patient underwent a pelvic angiogram that showed a network of communications between the left common femoral artery and common femoral vein, and the median sacral artery and left L5 lumbar artery. Embolization was performed in the left internal iliac artery using coils and Onyx. Next, left common iliac vein (CIV) occlusion was treated with balloon angioplasty, and two stents were inserted. Post-stenting venogram showed patent iliac veins and no collaterals. On follow-up, the patient's left leg edema decreased substantially, improving the patient's quality of life (Figure 5).

## Discussion

We searched the literature for similar cases. We found two case reports of patients with no preceding orthopedic surgery presenting with progressive leg swelling and were found to have AVF after clinical suspicion of DVT. One patient developed acute kidney injury due to venous infarction, and the other developed neurological deficits [4,5]. We found several similar case reports with a history of orthopedic surgery. A 40-year-old woman with a one-year history of right leg swelling following laminectomy and lumbar discectomy was initially diagnosed as DVT on a clinical basis and treated with anticoagulation. However, her symptoms persisted, and later she developed heart failure. The presence of AVF was confirmed, and she underwent vascular surgery complicated by ischemia requiring amputation. Post-operatively, her heart failure resolved [6]. A 91-year-old woman developed extensive left leg DVT following right hip fracture repair; however, her symptoms worsened despite anticoagulation, and she developed superficial ulcerations, cyanosis, and painful toes. She had excellent improvement following the coiling of AVF and stenting CIV [7]. Yuan et al. reported 24 cases of AVF diagnosed after initial diagnosis of DVT not related to previous surgery or trauma. All cases had severe stenosis or occlusion of left CIV, similar to our patient, raising suspicion of a causal relation between DVT and AVF [8].

The main feature common in the case reports we reviewed and our patient is the persistence or progression of symptoms despite adequate anticoagulation after initial diagnosis of DVT. Fortunately, this patient did not develop other complications seen in the reviewed case reports. Thus, lack of improvement with adequate anticoagulation should raise suspicion of alternative diagnoses such as AVF.

## Conclusions

Although DVT is one of the most common causes of leg swelling following lower extremity orthopedic surgery, we have to consider AVF, especially with worsening symptoms despite optimal standard therapy, presence of neurological deficits, heart failure, ulceration, cyanosis, or findings of an arterialized venous waveform on venous Doppler examination.
